# Editorial: CRISPR/CAS innovations: advancing precision and specificity in plant genetic engineering

**DOI:** 10.3389/fpls.2026.2012559

**Published:** 2026-10-21

**Authors:** Giuseppe Dionisio, Siddharth Tiwari, Phanikanth Jogam

**Affiliations:** 1 Department of Agroecology, Crop Genetics and Biotechnology, Aarhus University, Slagelse, Denmark; 2 Plant Tissue Culture and Genetic Engineering Lab, BRIC-National Agri-Food and Biomanufacturing Institute (BRIC-NABI), Department of Biotechnology, Ministry of Science and Technology, Government of India, Mohali, Punjab, India; 3 Multidisciplinary Research Unit, Kakatiya Medical College, Warangal, India

**Keywords:** CRiSPR/Cas, crop improvement, DNA-free genome editing, plant biotechnology, plant genome editing, precision genome engineering, ribonucleoprotein, virus-induced genome editing

Genome editing has transformed plant biotechnology from a discipline largely dependent on random genetic modification and lengthy breeding cycles into one increasingly capable of targeted and predictable genome engineering (Chen et al., 2019). Among the available technologies, CRISPR/Cas systems have become particularly influential because of their versatility, programmability, and applicability across plant species (Belhaj et al., 2013). Nevertheless, the transition from proof-of-concept editing to routine crop improvement requires more than efficient induction of mutations. Precision, specificity, delivery, regeneration, heritability, and the ability to obtain plants free of permanently integrated editing constructs are increasingly important considerations (Zhang et al., 2018).

This Research Topic was conceived to address these challenges. Its central objective was to explore technological advances capable of increasing the accuracy and reliability of CRISPR-mediated plant genome engineering while reducing unintended genetic alterations. The scope encompassed innovations in CRISPR/Cas systems, off-target prediction and detection, Cas protein engineering, guide RNA optimization, base and prime editing, high-throughput mutation analysis, and alternative strategies for delivering genome-editing reagents. Particular emphasis was placed on approaches that minimize prolonged nuclease activity or integration of Cas-expressing constructs, including ribonucleoprotein (RNP), protoplast-, nanoparticle-, and virus-mediated delivery. Collectively, such developments are essential for translating genome editing into sustainable crop improvement (Kocsisova and Coneva, 2023).

This principle is demonstrated clearly by Ferrero et al., who established an RNP-mediated genome-editing workflow in eggplant (*Solanum melongena*). By combining protoplast isolation, regeneration and transient delivery of CRISPR/Cas9 RNPs, the study provides a route toward editing without stable integration of CRISPR/Cas DNA. The recovery of edited plants, including biallelic events, represents an important technical advance for a crop in which regeneration remains a major bottleneck. More broadly, the study illustrates the close relationship between editing chemistry, delivery technology and plant regeneration: highly precise molecular reagents have practical value only when edited cells can ultimately be converted into viable plants.

DNA-free RNP delivery also provides a direct route from precision editing to crop quality traits. Chatani et al. used CRISPR/Cas9 RNPs delivered to soybean shoot apical meristems to target *Gly m 4-L1*, identifying this locus as a major determinant of Gly m 4 allergen accumulation in seeds. The resulting mutants showed absence of detectable Gly m 4 protein and markedly reduced IgE reactivity. This study therefore extends the significance of precision genome engineering beyond agronomic performance to food quality and potentially human nutrition, while demonstrating the utility of DNA-free editing for generating targeted crop phenotypes.

An alternative and innovative strategy to the delivery bottleneck is provided by Tibebu et al., who established Tobacco rattle virus (TRV)-mediated virus-induced genome editing (VIGE) in groundcherry (*Physalis grisea*). Rather than delivering the complete CRISPR/Cas9 machinery, this approach exploits the capacity of TRV to replicate and move systemically through infected plants, carrying sgRNAs to tissues distant from the initial inoculation site. Importantly, the sgRNAs were fused to a truncated *Arabidopsis thaliana* FLOWERING LOCUS T (FT) sequence that enhances their mobility between cells, thereby facilitating access to meristematic and germline-associated tissues and enabling recovery of heritable edits. Because TRV cannot stably accommodate the relatively large *Streptococcus pyogenes* Cas9 coding sequence, VIGE in this configuration requires the prior generation of transgenic groundcherry plants constitutively expressing Cas9. Once such parental Cas9 lines are established, however, different mobile sgRNAs can subsequently be introduced by viral infection without repeating transformation and tissue culture for every target. The authors demonstrated highly efficient somatic editing of *phytoene desaturase* (*PDS*) (approximately 80–95%) and recovery of heritable mutations, as well as editing of the domestication gene *CLAVATA1* (*CLV1*), producing altered floral architecture and multilocular fruits. Remarkably, efficient mono-, bi-, tri- and tetra-allelic editing was achieved in an unexpectedly tetraploid Cas9 line. Thus, the principal innovation of this work lies in combining a reusable Cas9-expressing genetic background with the systemic mobility of virus-delivered sgRNAs, providing a rapid and flexible platform for generating heritable genetic diversity and potentially accelerating the domestication and improvement of underutilized crops (**Figure 1**).

**Figure 1:**
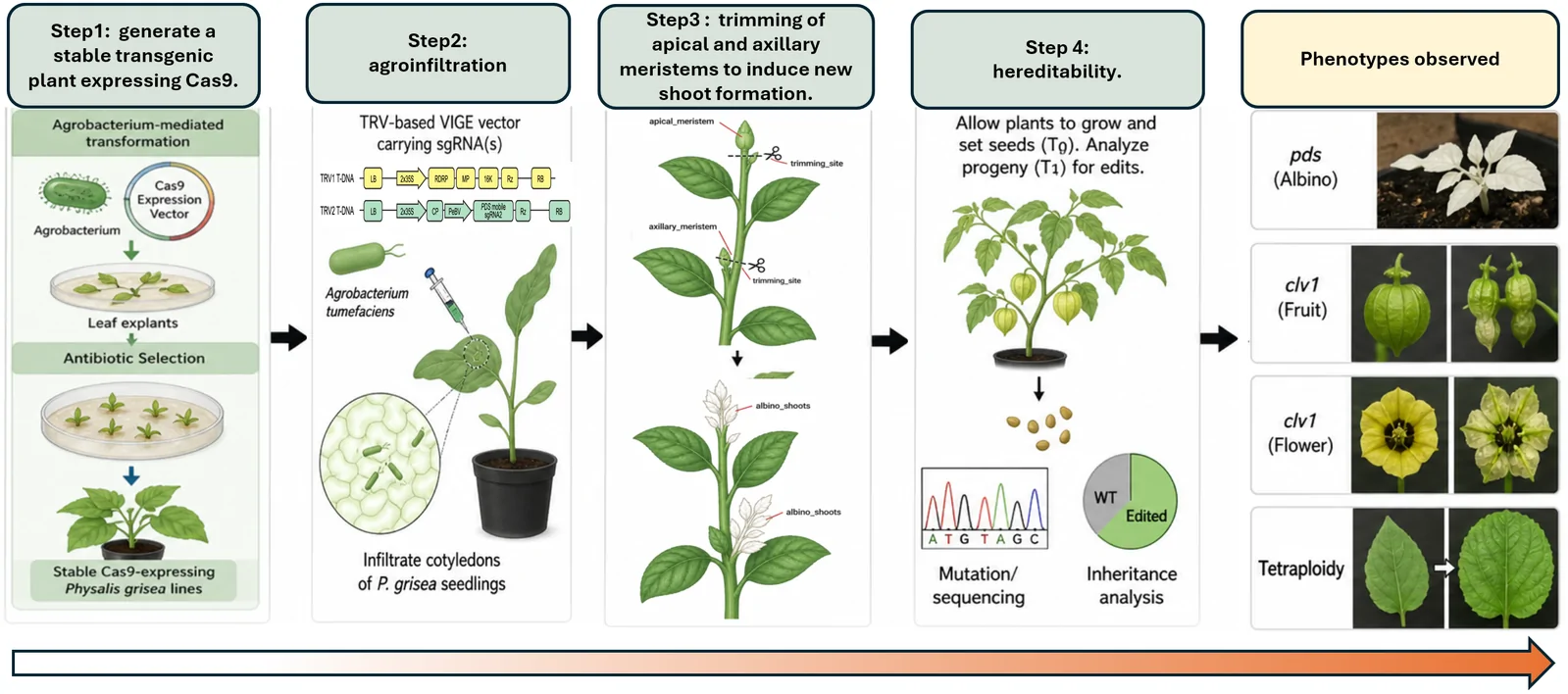
Workflow for virus-induced genome editing (VIGE) in groundcherry (*Physalis grisea*). Stable Cas9-expressing plants are first generated by *Agrobacterium*-mediated transformation and subsequently agroinfiltrated with Tobacco rattle virus (TRV) vectors carrying mobile sgRNAs. Trimming of apical and axillary meristems promotes the emergence of edited shoots, which are allowed to flower and set seed for assessment of mutation inheritance. The approach enables efficient somatic and heritable editing, as demonstrated by the *phytoene desaturase* (*PDS*) and *CLAVATA1* (*CLV1*) mutant phenotypes and recovery of edited progeny with high mutation frequency.

The importance of extending CRISPR technologies to locally important crops was further demonstrated by Kalungi et al.. Their establishment of efficient CRISPR/Cas9 editing of *PDS* in East African highland banana represents an enabling step toward genome editing of this major regional staple. High editing efficiencies were obtained in both a local cultivar and a hybrid genotype, despite the complexity of the triploid banana genome. Although this study employed *Agrobacterium*-mediated transformation, it establishes the biological and technical foundation upon which more advanced editing and delivery strategies can subsequently be developed for traits associated with disease resistance, climate resilience, quality and productivity.

In another study, Pinski et al. established a CRISPR/Cas9-based genome-editing system in common buckwheat (*Fagopyrum esculentum*) to overcome distyly-associated self-incompatibility. The *S-locus* early flowering 3 (*FeS-ELF3*) gene was identified as a major genetic determinant controlling floral morphs and self-incompatibility in common buckwheat. Targeted knockout of *FeS-ELF3* generated self-compatible, long-homostylous plants, demonstrating the potential of genome editing to overcome reproductive barriers associated with distyly. The edited line exhibited reduced plant height and inflorescence length but showed no apparent reduction in seed yield under greenhouse conditions. However, the generation and evaluation of additional independently edited lines, along with field testing, will be important for validating these phenotypic effects and determining their agronomic stability. Taken together, these contributions reveal an important evolution in plant genome engineering. The question is no longer simply whether CRISPR can edit a plant genome, but how editing can be made precise, transient, heritable, genotype-accessible and compatible with practical breeding. RNP delivery can restrict nuclease exposure and avoid stable integration of editing constructs; viral vectors can bypass some barriers imposed by transformation and tissue culture; and improved regeneration systems can convert precisely edited single cells into whole plants. At the same time, successful editing of polyploid and vegetatively propagated crops demonstrates that genome editing is becoming applicable to biological contexts that have historically been challenging for conventional breeding.

Future progress will require improved Cas protein variants, optimized guide design, base and prime editors, computational prediction of editing outcomes, sensitive off-target detection, and innovative delivery technologies. These advances should increasingly be considered components of an integrated precision-engineering pipeline rather than independent developments. Equally important will be robust phenotypic validation and regulatory frameworks that distinguish targeted genome editing from conventional transgenic modification, where scientifically appropriate (Duensing et al., 2018).

The contributions to this Research Topic therefore highlight both the maturity of CRISPR/Cas technology and the challenges that remain. Precision ultimately encompasses more than nucleotide-level specificity: it includes precise reagent delivery, precise control of editing activity, precise recovery of edited genotypes and precise translation of genetic changes into useful agricultural phenotypes. Continued integration of molecular engineering, delivery science, plant regeneration, genomics and breeding will be essential to realizing the full potential of CRISPR technologies in developing resilient, productive and sustainable crops (Cardi et al., 2023).
